# An Unusual Case of Familial Adenomatous Polyposis Presenting with Gout and Jaundice

**DOI:** 10.7759/cureus.1475

**Published:** 2017-07-15

**Authors:** Anna K Lawless, Ravi Huilgol, Christopher J Young

**Affiliations:** 1 Junior Medical Officer, Royal Prince Alfred Hospital; 2 Vascular Surgery, St. Vincent's Hospital; 3 Colorectal Unit, Royal Prince Alfred Hospital

**Keywords:** adenoma, adenomatous polyposis coli, common bile duct neoplasms, gout, ampulla of vater, colectomy, familial adenomatous polyposis

## Abstract

We highlight an unusual case of familial adenomatous polyposis (FAP) presenting initially with gout, jaundice and a periampullary carcinoma. This case may be of interest to clinicians involved in the diagnosis and management of FAP and follow-up of patients after surgical resection.

## Introduction

Familial adenomatous polyposis (FAP) is a precancerous syndrome of autosomal dominant inheritance, affecting approximately one in 10,000 individuals and characterized by multiple colorectal adenomas as well as extracolonic manifestations [1]. Up to 100% of these patients will develop adenomas of the duodenum and periampullary region, with a high likelihood of progression to adenocarcinoma if left untreated [2]. Advances in genetic screening technology have decreased the mortality rates of colorectal cancer in this condition, such that duodenal cancer and desmoid tumors now represent the main cause of death [3]. Informed consent statement was obtained for this study.

## Case presentation

A 33-year-old male presented with an inflamed right first metatarsophalangeal joint of 24 hours duration, with no history of trauma. He had no significant medical history, but on questioning revealed a three-month history of intermittent pruritus and dark urine. There was no history of a change in bowel habit or bleeding per rectum and no family history of polyps or colorectal cancer.

**Figure 1:**
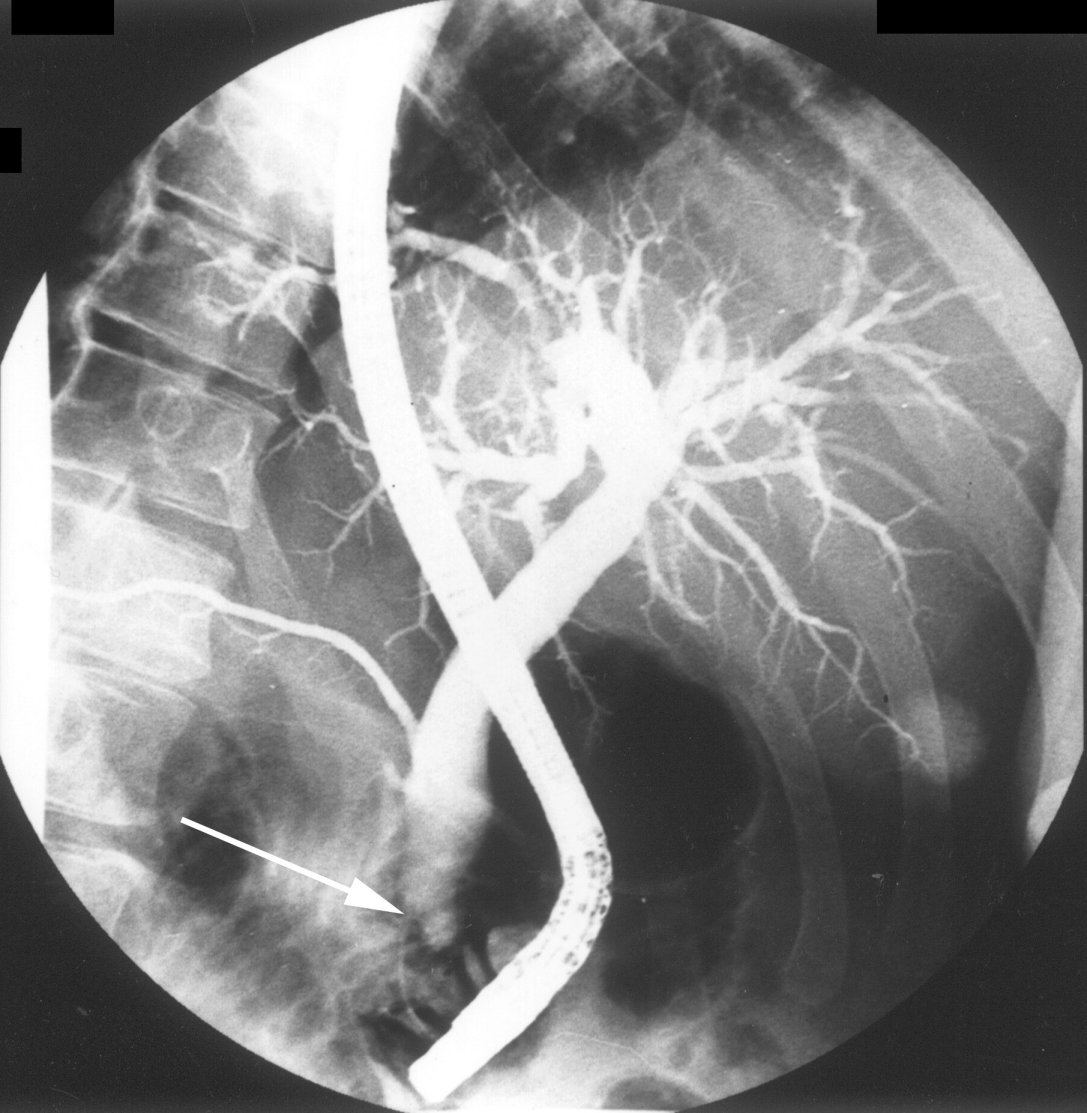
Endoscopic retrograde cholangiopancreatography demonstrates an intraluminal lesion at the duodenal ampulla of Vater

Laboratory tests were largely unremarkable, apart from a serum uric acid of 0.44 mmol/L, gamma-glutamyl transferase test (GGT) 1878 U/L, alkaline phosphatase test (ALP) 1143 U/L, aspartate aminotransferase (AST) 426 U/L, 615 78 U/L, and a bilirubin of 72 mmol/L. A diagnosis of gout was made and managed with a simple analgesic. An upper abdominal ultrasound showed common bile duct dilatation. Endoscopic retrograde cholangiopancreatography (ERCP) subsequently demonstrated an intraluminal lesion at the duodenal ampulla of Vater and biopsies demonstrated a villous adenoma (Figure 1).

**Figure 2:**
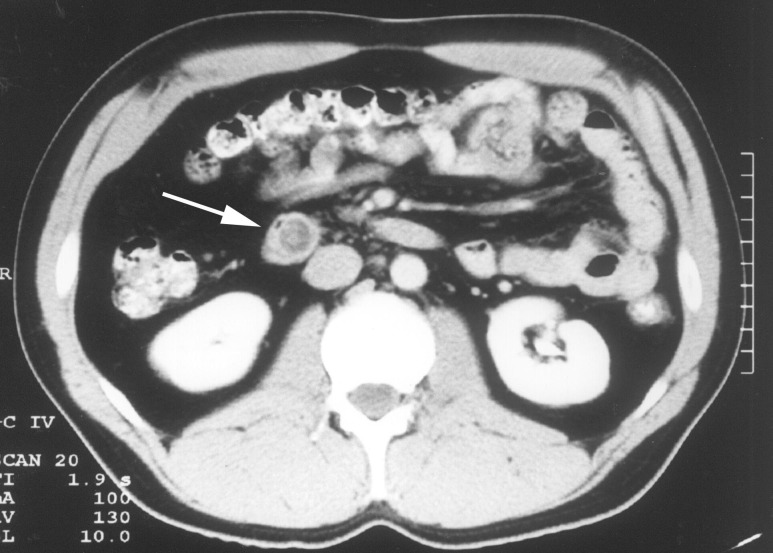
Abdominal computed tomography (CT) scan demonstrates an ampullary mass

Rigid sigmoidoscopy revealed multiple rectosigmoid polyps, and computed tomography (CT) scan of the abdomen confirmed the ampullary mass, with no evidence of metastatic spread (Figure 2).

On referral to a tertiary institution, acute gout had resolved, but the patient had become mildly jaundiced. Colonoscopy and biopsy revealed hundreds of pedunculated and sessile tubulovillous and tubular adenomas involving the colon and rectum to the anorectal junction.

Radical pancreaticoduodenectomy and cholecystectomy were performed due to the high chance of underlying malignancy. Histology revealed an adenocarcinoma arising from a villous adenoma at the duodenal ampulla and multiple duodenal adenomas, highlighting the limitations of ampullary polyp biopsy. Three months later, the patient was readmitted and underwent total colectomy and ileorectal anastomosis. A pelvic pouch procedure had been planned, but the pancreaticoduodenectomy made it not possible to bring the terminal ileum down to the pelvic floor for reconstruction.

The patient made a good recovery from this operation and was discharged home on the tenth postoperative day. At four year follow-up, there was no recurrence of the periampullary carcinoma and the patient's rectal polyps were well controlled with six-monthly sigmoidoscopic ablation thereafter.

## Discussion

To our knowledge, this is the first case of FAP presenting with gout and jaundice reported in the literature. The presence of gout may have been mere coincidence or precipitated by hyperuricemia caused by increased nucleotide catabolism due to underlying malignancy [4]. This case is unique due to the initial presentation with adenocarcinoma of the duodenal ampulla of Vater, necessitating treatment. Typically, upper gastrointestinal tract (GIT) pathology in FAP develops after lower GIT disease, but our patient developed and required treatment of his upper GIT disease first [2].

Upper GIT manifestations of FAP will affect up to 100% of patients, and their occurrence may be related to the carcinogenic properties of bile [5]. The incidence and severity of periampullary neoplasia have a genetic basis, with adenomatous polyposis coli (APC) mutations linked to the phenotypic expression of gastric and duodenal polyps [6]. These adenomas progress slowly by increasing size and dysplasia toward cancer, but it remains difficult to predict which adenomas will progress to adenocarcinoma.

Upper GIT screening is advocated by most centers, due to the high incidence and increasing severity of duodenal adenomatosis with age [2]. Endoscopic resection is the preferred treatment, although recurrence rates are high [2, 7]. Due to their slow progression, prophylactic surgery for duodenal adenomas is not advocated, although there is some evidence for the prophylactic use of cyclooxygenase inhibitors [2, 7].

For malignant duodenal polyps, pancreas-sparing duodenectomy or pancreaticoduodenectomy are recognized options [7]. Our patient underwent pancreaticoduodenectomy due to the high chance of underlying malignancy. This case illustrates the inaccuracy of ampullary polyp biopsy in detecting underlying malignancy, which may be the case in up to one-third of biopsy results [8].

## Conclusions

This case highlights an unusual presentation of familial adenomatous polyposis (FAP) presenting with gout and jaundice, and with upper gastrointestinal tract (GIT) disease requiring treatment before lower GIT disease. Pancreaticoduodenectomy was performed and the mass was found to be malignant despite the original ampullary polyp biopsy demonstrating adenoma. This case may be of interest to clinicians in the diagnosis and management of FAP, in particular, the unusual presentation, and the inaccuracy of ampullary polyp biopsy in detecting underlying malignancy in high-risk patients.
